# Lipid Dysregulation in Tangier Disease: A Case Series and Metabolic Characterization

**DOI:** 10.1210/clinem/dgaf131

**Published:** 2025-03-03

**Authors:** Georg Semmler, Clemens Baumgartner, Matthäus Metz, Sophie Gensluckner, Hansjörg Habisch, Hannah Hofer, Winfried März, Felix Offner, Andreas Völkerer, Oleksandr Petrenko, Bernhard Wernly, Sophie Draxler-Dworzak, Manuela Neyer, Charlotte Nigmann, Susanne Greber-Platzer, Harald Esterbauer, Tobias Madl, Elmar Aigner, Thomas Scherer, Christian Datz

**Affiliations:** Division of Gastroenterology and Hepatology, Department of Medicine III, Medical University of Vienna, Vienna 1090, Austria; Centre for Liver Research, Department of Gastroenterology and Hepatology, Odense University Hospital, Odense 5000, Denmark; Institute of Clinical Research, Faculty of Health Sciences, University of Southern Denmark, Odense 5230, Denmark; Division of Endocrinology and Metabolism, Department of Medicine III, Medical University of Vienna, Vienna 1090, Austria; Clinical Department of Nephrology and Dialysis, Department of Medicine III, Medical University of Vienna, Vienna 1090, Austria; Division of Endocrinology and Metabolism, Department of Medicine III, Medical University of Vienna, Vienna 1090, Austria; Synaptic Transmission in Energy Homeostasis Group, Max Planck Institute for Metabolism Research, Cologne 50931, Germany; First Department of Medicine, Paracelsus Medical University Salzburg, Salzburg 5020, Austria; Division of Medical Chemistry, Otto Loewi Research Center for Vascular Biology, Immunology and Inflammation, Medical University of Graz, Graz 8010, Austria; Department of Internal Medicine, General Hospital Oberndorf, Teaching Hospital of the Paracelsus Medical University Salzburg, Oberndorf, Salzburg 5110, Austria; Department of Internal Medicine V, Medical Faculty Mannheim, Heidelberg University, Mannheim 68167, Germany; Clinical Institute of Medical and Chemical Laboratory Diagnostics, Medical University of Graz, Graz 8010, Austria; SYNLAB Academy, SYNLAB Holding Deutschland GmbH, Augsburg and Mannheim 86156, Germany; Department of Pathology, Academic Teaching Hospital Feldkirch, Feldkirch 6800, Austria; Department of Internal Medicine, General Hospital Oberndorf, Teaching Hospital of the Paracelsus Medical University Salzburg, Oberndorf, Salzburg 5110, Austria; Division of Gastroenterology and Hepatology, Department of Medicine III, Medical University of Vienna, Vienna 1090, Austria; Department of Laboratory Medicine, Medical University Vienna, Vienna 1090, Austria; Ukrainian Institute for Systems Biology and Medicine, Kyiv 03143, Ukraine; First Department of Medicine, Paracelsus Medical University Salzburg, Salzburg 5020, Austria; Department of Internal Medicine, General Hospital Oberndorf, Teaching Hospital of the Paracelsus Medical University Salzburg, Oberndorf, Salzburg 5110, Austria; Division of Pediatric Pulmonology, Allergology and Endocrinology, Department of Pediatrics and Adolescent Medicine, Medical University of Vienna, Vienna 1090, Austria; Division of Pediatric Pulmonology, Allergology and Endocrinology, Department of Pediatrics and Adolescent Medicine, Medical University of Vienna, Vienna 1090, Austria; Division of Pediatric Pulmonology, Allergology and Endocrinology, Department of Pediatrics and Adolescent Medicine, Medical University of Vienna, Vienna 1090, Austria; Division of Pediatric Pulmonology, Allergology and Endocrinology, Department of Pediatrics and Adolescent Medicine, Medical University of Vienna, Vienna 1090, Austria; Department of Laboratory Medicine, Medical University Vienna, Vienna 1090, Austria; Division of Medical Chemistry, Otto Loewi Research Center for Vascular Biology, Immunology and Inflammation, Medical University of Graz, Graz 8010, Austria; BioTechMed Graz, Graz 8010, Austria; First Department of Medicine, Paracelsus Medical University Salzburg, Salzburg 5020, Austria; Division of Endocrinology and Metabolism, Department of Medicine III, Medical University of Vienna, Vienna 1090, Austria; Department of Internal Medicine, General Hospital Oberndorf, Teaching Hospital of the Paracelsus Medical University Salzburg, Oberndorf, Salzburg 5110, Austria

**Keywords:** Tangier disease, ABCA1, cholesterol, high-density lipoprotein, HDL

## Abstract

**Context:**

Tangier disease (TD) is a rare, autosomal recessive genetic disorder associated with a deficiency in cellular cholesterol export leading to cholesterol accumulation in peripheral tissues. With approximately 150 described cases, the disease is significantly understudied, and the clinical presentation appears to be heterogenous.

**Objective:**

To investigate the phenotype and lipid metabolism in TD.

**Design:**

Multicenter cohort study.

**Patients:**

Four patients with TD.

**Main Outcome Measures:**

Nuclear magnetic resonance (NMR)-based lipidomic and metabolomic analyses were performed in patients with TD and healthy controls.

**Results:**

While showing similar laboratory patterns with respect to high-density lipoprotein (HDL) depletion, the clinical presentation of 4 TD patients was heterogenous with 2 patients diagnosed at 47 and 72 years having predominantly gastrointestinal and neurological phenotypes. Two previously undescribed variants (c.2418G > A, c.5055.del) were reported.

Apart from pathognomonic changes in HDL composition, NMR spectroscopy revealed an increased abundance of very low-density lipoprotein (VLDL) with higher total lipid and cholesterol concentrations, pointing toward an impaired clearance of triglyceride-rich lipoproteins. Increased triglyceride-rich intermediate-density lipoprotein supports impaired hepatic lipase activity, together with a cholesteryl ester transfer protein-mediated increase in low-density lipoprotein (LDL)-triglycerides at higher abundance of large LDL subtypes and decreased small dense LDL.

The lipid composition of HDL particles and LDL-1/LDL-4 remained the strongest differentiating factors as compared to healthy controls.

**Conclusion:**

Clinical phenotypes of TD can be heterogeneous including gastrointestinal and neurological manifestations. Impaired triglyceride-rich lipoprotein clearance and hepatic lipase activity could be a pathophysiological hallmark of TD.

Tangier disease (TD) is a rare, autosomal recessive genetic disorder associated with a deficiency in cellular cholesterol and phospholipid export mediated by the ATP binding cassette transporter A1 (*ABCA1*) (1, 2). With an estimated global prevalence of 1 in 640,000 based on allele frequencies of the responsible *ABCA1* gene (3), a rare characterization with approximately 150 published cases worldwide strongly underestimates the real numbers of patients and fails to describe the complete clinical picture (1). Although *ABCA1* is ubiquitously expressed in the human body, its functions are tissue-dependent and include the assembly of high-density lipoproteins (HDL) in hepatocytes, intestinal enterocytes, and adipocytes, as well as mediating the cholesterol efflux out of peripheral macrophages (first step of reverse cholesterol transport from the periphery to the liver) (4-6). Specifically, loss-of-function variants in *ABCA1* lead to intracellular cholesterol accumulation in the reticuloendothelial system and peripheral macrophages (“foam cells”), which may infiltrate various organs and essentially contribute to the development of atherosclerosis (7). Characteristic laboratory findings include extremely low levels of HDL and apolipoprotein-A (Apo-A)-I and elevated triglycerides and thrombocytopenia. Typical clinical findings include orange-colored tonsils, peripheral neuropathy, and hepatosplenomegaly, with cardiovascular diseases developing in ∼25% of subjects (prevalence 35-65 years: ∼45%) (1, 8-11). Recently, a gastrointestinal manifestation with orange-brown focal deposits in the intestinal mucosa resulting in a diffuse infiltration of the colonic mucosa with foam cells has been described (12, 13).

Plasma metabolomics represents a novel approach to characterize metabolic alterations in a holistic way (14). While high-throughput approaches using nuclear magnetic resonance (NMR) spectroscopy exist, metabolic alterations have not been further investigated in patients with TD.

As disease prevalence might be much higher and clinical presentation more heterogenous, the aim of this study was to characterize clinical and laboratory findings in 4 male patients with TD at different ages and investigate their metabolic profile using NMR-based lipidomics and metabolomics.

## Materials and Methods

### Patients and Design

This is a retrospective case series of 4 patients with TD presenting in 3 different hospitals in Austria. Patients were characterized at the first presentation including clinical and laboratory assessment with further symptom-oriented examinations. All blood samples including those used for NMR spectroscopy were drawn in fasting condition.

### NMR Spectroscopy

Plasma samples from all patients as well as 10 healthy volunteers [previously described in (15)] were used to study plasma lipids and metabolites by high-throughput NMR spectroscopy (method 1), as previously described (16). Additionally, plasma samples of patients #2 and #3 as well as 13 healthy volunteers have been separately tested by Nightingale Health Plc. (Helsinki, Finland; method 2) (14). A detailed methodology can be found in the supplemental materials (17).

### Ethics

The study was approved by the local ethics committee of the Medical University of Vienna (2371/2024) and performed in conformity with the Declaration of Helsinki. Written informed consent for publication of their clinical details was obtained from all patients.

### Statistical Analysis

Statistical analyses were performed using R 4.4.1 (R Core Team, R Foundation for Statistical Computing, Vienna, Austria). Metabolites were numerically compared between TD patients and healthy controls and reported as median and range. Principal component analysis (PCA) including absolute metabolite metrics (i.e, excluding percentages, size, and ratios) was performed using the “PCAtools” package to assess variable importance for differentiation between TD patients and healthy controls. Differential metabolite abundance was tested using the LIMMA method (“limma” package) and with Benjamini-Hochberg's adjustment of *P*-values. *P*_adjusted_ of < .05 and log fold change ≥ |0.5| were used to identify significantly dysregulated metabolites. Metabolites were log-transformed and scaled prior to further analyses.

## Results

### Clinical Presentation

Four male patients were diagnosed with TD at the age of 22 (patient #1) and 72 (patient #4) after low HDL was detected in routine blood tests and at the age of 47 during the workup of chronic hyperferritinemia and cardiomyopathy of unknown origin (patient #3). Patient #2 (sibling of patient #1) was diagnosed at the age of 19 following family testing of his brother. Clinical presentation was highly heterogeneous (Table 1), including occasional sensory dysesthesia or hypesthesia in patients #1 and #2, unclear cardiomyopathy and marked hyperferritinemia in patient #3, and progressive neurodegenerative dementia with evidence of amyloid disposition in relation to large intracranial arteries on magnetic resonance imaging in patient #4. All patients had splenomegaly and 3 of 4 had a history of tonsillectomy due to reported orange-colored tonsils in 2 of them. Importantly, patient #3 had no evidence of coronary artery disease (CAD) on coronary angiography despite unclear heart failure with focal fibrosis on cardiac magnetic resonance imaging. Similarly, patient #4 did not report angina or a history of CAD. Also, neither had evidence of atherosclerosis including normal carotid ultrasound. However, both had severe hepatic steatosis as assessed by transient elastography, the latter with evidence of advanced fibrosis. In contrast to the clinical presentation, standard lipid parameters were similar including thrombocytopenia (due to hypersplenism), low low-density lipoprotein (LDL) levels (7-50 mg/dL), elevated triglycerides in all except patient #2 (targeted family testing in 19-year-old), and undetectable HDL and ApoA-I (Table 2). Notably, patients #3 and #4 had chronic hyperferritinemia (2493 and 686 ng/mL) that persisted during the subsequent years. Genetic testing confirmed homozygous loss-of-function variants in *ABCA1* (c.2418G > A in patients #1 and #2, c.679C > T in patient #3, and c.5055del in patient #4, all regarded as American College of Medical Genetics class 5 (pathogenic). Notably, only c.679C > T has been previously published (18). A detailed presentation of all cases is presented in the supplemental materials (17).

**Table 1.** Clinical characterization of 4 male patients with TD at first presentation

Parameter	Patient #1*^a^*	Patient #2*^a^*	Patient #3	Patient #4
Age (years)	22	19	47	72
BMI (kg/m²)	27.8	27.4	25.6	27.0
Initial symptom	Low HDL	Family testing	Chronic hyperferritinemia	Low HDL
Country of origin	Turkey	Turkey	Austria	Austria
Family history of premature CVD	Uncle 55a	Uncle 55a	No	No
Smoking	Yes	No	Yes	History of
Arterial hypertension	No	No	Yes	Yes
Splenomegaly (cm)	17.2	15.0	13.3	19.0
Peripheral neuropathy	Subjective hypesthesia	Subjective hypesthesia	Yes	No
Ophthalmological	Normal	Normal	Corneal dystrophy	—
Osteoporosis	Osteopenia	—	Yes	—
Heart failure	No	No	HFmrEF*^b^*	—
Orange tonsils	Yes, history of tonsillectomy	History of tonsillectomy due to chronic tonsillitis	No	Unclear, history of tonsillectomy
Other symptoms	—	—	Gastrointestinal manifestation, hyperpigmentation lower extremities, chronic lymphedema	Progredient neurodegenerative dementia with amyloid disposal
Transthoracal echocardiography	Normal	Normal	HFmrEF, dilated left artrium + ventricle	Left-ventricular hypertrophy + dilated left atrium
Transient elastography (liver)	—	—	LSM 5.6 kPa (F0), CAP 336 dB/m (S3)	LSM 11.1 kPa (F3/4), CAP 348 dB/m (S3)
Coronary angiography	—	—	Normal	—
Carotid ultrasonography	Normal	Normal	Normal	Normal
Nerve conduction velocity	Normal	Normal	Not done (but neurologic status from neurologist typical with polyneuropathy)	Not done (no suspicion from neurologist)

Abbreviations: BMI, body mass index; CAP, controlled attenuation parameter; CVD, cardiovascular disease; HDL, high-density lipoprotein; HFmrEF, heart failure with midrange ejection fraction; LSM, liver stiffness measurement; TD, Tangier disease.

^*a*^Siblings.

^*b*^The patient developed clinical decompensation within the subsequent 2 years.

**Table 2.** Laboratory characterization of four male patients with TD at first presentation

Parameter	Patient #1*^a^*	Patient #2*^a^*	Patient #3	Patient #4	Reference
White blood cells (G/L)	5.14	8.1	5.2	4.1	4-10
Hemoglobin (g/dL)	15.2	14.9	13.9	**11.3**	13.5-18.0
Mean corpuscular volume (fl)	88.1	89.4	97.9	**106.7**	78-98
Mean corpuscular hemoglobin (pg)	30.6	30.8	32.3	**35.9**	27-33
Platelet count (G/L)	**113**	**126**	**103**	**77**	150-350
Cholesterol (mg/dL)	51	58	65	53	<200
HDL (mg/dL)	**<3**	**<3**	**<5**	**<3**	>40
LDL (mg/dL)	37	50	31	7	<116
Triglycerides (mg/dL)	**158**	77	**188**	**468**	<150
ApoA-I (mg/dL)	**<20**	**<20**	**<20**	**<20**	>125
ApoB (mg/dL)	73	78	80	70	<100
Lipoprotein(a) (nmol/L)	<7	<7	—	<7	<75
C-reactive protein (mg/dL)	0.13	<0.03	**3.28**	0.2	<0.5
Ferritin (ng/mL)	28	41	**2493**	**686**	21.8-274.7
Transferrin (mg/dL)	369	325	207	210	174-364
Transferrin saturation (%)	21	12	22	28	15-45
NT-proBNP (pg/mL)	—	—	13	**202**	<100
Mutation (homozygous)	c.2418G > A	c.2418G > A	c.679C > T, p.(Arg227Ter)	c.5055del, p.(Lys1685AsnfsTer9)	
ACMG classification	5 –pathogenic	5 –pathogenic	5 –pathogenic	5 – pathogenic	

Bold values indicate that values outside the normal range (reference).

Abbreviations: ACMG, American College of Medical Genetics; ApoA-I, apolipoprotein A1; ApoB, apolipoprotein B; HDL-C, high-density lipoprotein cholesterol; LDL-C, low-density lipoprotein; NT-proBNP, N-terminal pro–B-type natriuretic peptide; TD, Tangier disease.

^*a*^Siblings.

### Gastrointestinal Manifestation of Patient #3

**Figure 1.**
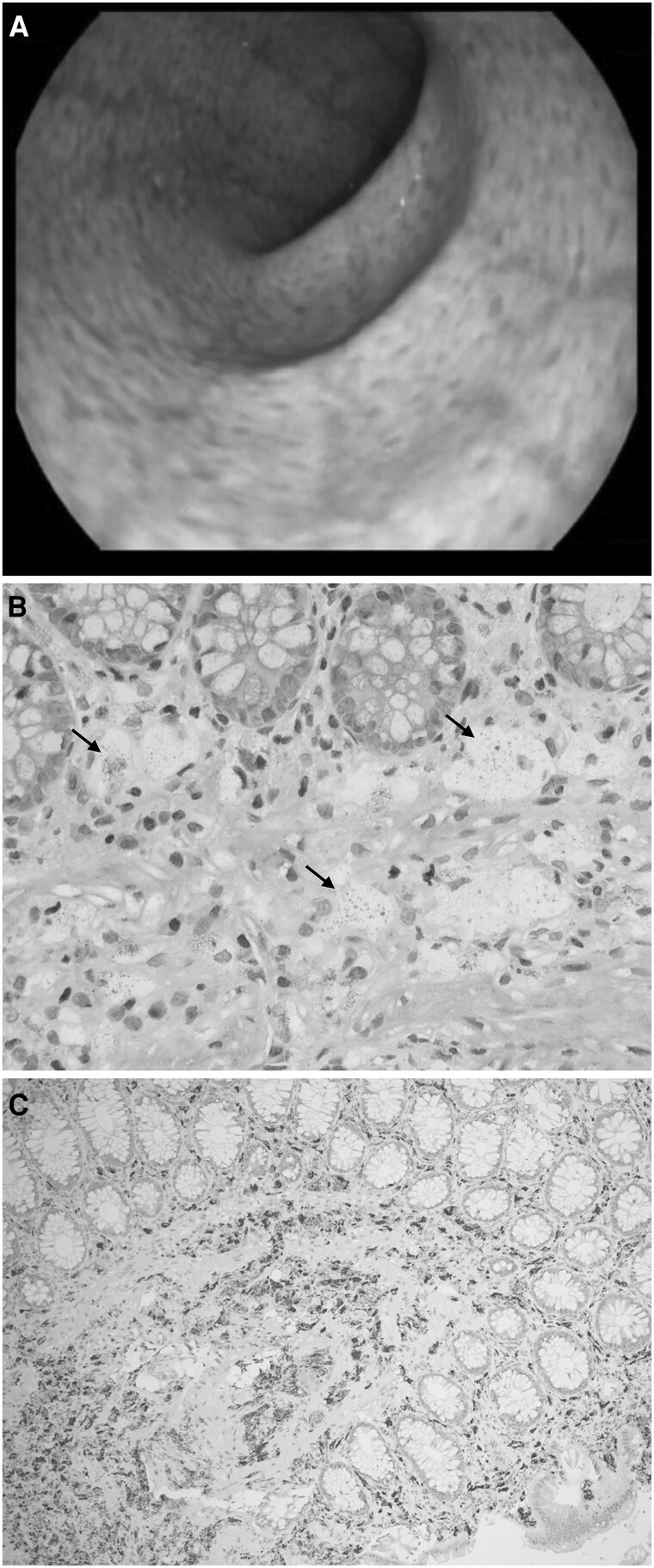
Endoscopic images from colonoscopy of patient #3 showing edematous mucosa with hyperpigmentation (A) and histological images from the colonic biopsies showing focal aggregation of macrophages with large foamy cytoplasm and granular PAS positivity (arrows), both in the mucosa and submucosa: (B) PAS staining; (C) immunohistochemistry for CD68 marking macrophages (dark grey). Abbreviation: PAS, periodic acid-Schiff.

Surprisingly, patient #3 presented with a pronounced gastrointestinal manifestation discovered on combined esophagoduodenoscopy with colonoscopy to rule out gastrointestinal malignancy. The most remarkable findings were hyperpigmentation and edematous thickening of the colonic mucosa in all segments as well as irregular duodenitis (Fig. 1A). Histological evaluation revealed pronounced focal infiltration of the mucosa and the submucosa by CD68-positive foam cells [Fig. 1B and 1C, Supplemental Fig. S1 (17)] and several polyps formed predominantly by foam cells with iron deposition [Supplementary Figs. S2 and 3 (17)].

### Pedigrees of First-degree Relatives

Specifically, all first-degree relatives that were available and consented to further testing were genotyped for the described variants, and their lipid profiles were analyzed [Supplementary Fig. S4, Supplementary Table 1 (17)]. Of note, all relatives of patients #1 + 2 were heterozygous for the c.2418G > A loss-of-function variant with associated reduced HDL levels. Similarly, the son of patient #3 also carried the heterozygous c.679C > T variant with a similar lipid profile as the relatives of patients #1 + 2.

### Characterization of Lipid Metabolism

**Figure 2.**
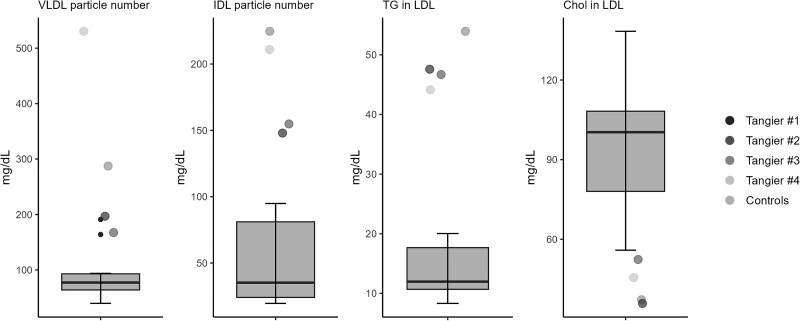
Box plots for graphical display of relevant lipid metabolites compared between TD patients (n = 4) and healthy controls (n = 10): VLDL particle number, IDL particle number, triglyceride concentration in LDL, and cholesterol in LDL box plots represent median and IQR with whiskers indicating 1.5*IQR. Absolute values are listed in Table 3. Abbreviations: IDL, intermediate-density lipoprotein; IQR, interquartile range; LDL, low-density lipoprotein; TD, Tangier disease; TG, triglycerides; TRL, triglyceride-rich lipoprotein; VLDL, very low density lipoprotein.

We performed NMR spectroscopy to characterize lipid metabolism in TD patients and compared these results with 10 healthy controls. Results are summarized in Table 3, Supplementary Table 2, and Fig. 2, including lipoprotein subclasses and their lipid composition (17). In general, plasma lipid profiles were profoundly altered in TD including pathognomonic findings of extremely low ApoA-I and an increased abundance of very low density lipoprotein (VLDL) and especially intermediate-density lipoprotein (IDL) particles. The total lipid concentration was increased in VLDL and IDL while it was decreased in LDL and HDL particles. At the same time, IDL and LDL triglyceride concentrations were markedly increased, while cholesterol, free cholesterol, and phospholipid content in LDL and HDL particles were decreased.

**Table 3.** Lipid NMR spectroscopy of 4 patients with TD compared to 10 healthy controls as well as 1 heterozygous relative

	Parameter	Unit	TD (n = 4), median [range]	TD patients #1-#3, median [range]	Healthy controls (n = 10), median [range]	Heterozygous relative (n = 1)	Direction (all)	Direction (#1-#3)
Overall concentrations	Cholesterol	mg/dL	76 [73.3-122]	75.7 [73.3-76.3]	169 [127-207]	219	↓↓	↓↓
	Total triglycerides	mg/dL	139 [96.3-461]	132 [96.3-145]	66.3 [38.3-165]	42.0	↑	↑
	ApoA-I	mg/dL	23.0 [18.6-64.9]	20.2 [18.6-25.7]	131 [114-154]	127	↓↓	↓↓
	ApoA-II	mg/dL	13.4 [11.9-34.6]	12.7 [11.9-14.1]	31.9 [28.2-34.1]	35.6		↓↓
	ApoB-100	mg/dL	71.1 [62.9-106]	67.8 [62.9-74.5]	65.8 [45.1-98.0]	35.6		
	ApoB-100/ApoA-I	Ratio	3.97 [2.48-4.37]	4.05 [2.48-4.37]	1.69 [1.00-3.56]	0.91	↑	↑
	LDL-cholesterol/HDL-cholesterol	Ratio	2.90 [1.63-3.99]	3.35 [2.45-3.99]	0.47 [0.33-0.84]	3.72	↑↑	↑↑
Lipoproteins	Total particle number	nmol/L	1290 [1140-1920]	1230 [1140-1350]	1200 [819-1780]	2100		
	VLDL particle number	nmol/L	242 [167-530]	197 [167-287]	77.3 [39.7-191]	39.5	↑	↑
	IDL particle number	nmol/L	183 [148-225]	155 [148-225]	35.2 [19.5-94.9]	146	↑↑	↑↑
	LDL particle number	nmol/L	1100 [1020-1400]	1050 [1020-1160]	1110 [684-1570]	2050		
	LDL-1 particle number	nmol/L	334 [220-380]	348 [320-380]	169 [109-303]	271	↑	↑↑
	LDL-2 particle number	nmol/L	303 [46.3-337]	329 [276-337]	176 [103-272]	237		↑↑
	LDL-3 particle number	nmol/L	141 [53.6-171]	151 [131-171]	150 [51.0-289]	346		
	LDL-4 particle number	nmol/L	0 [0-16.9]	0 [0-16.9]	126 [48.7-276]	460	↓↓	↓↓
	LDL-5 particle number	nmol/L	103 [35.7-232]	80.0 [35.7-126]	138 [91.6-327]	444		
	LDL-6 particle number	nmol/L	151 [73.1-1040]	121 [73.1-180]	228 [155-540]	325		
	HDL ApoA-I	mg/dL	29.6 [19.6-60.0]	26.7 [19.6-32.5]	132 [111-157]	126	↓↓	↓↓
	HDL ApoA-II	mg/dL	16.6 [15.8-36.3]	16.1 [15.8-17.1]	32.2 [29.5-34.4]	36.3		↓↓
	VLDL ApoB-100	mg/dL	13.3 [9.20-29.2]	10.8 [9.20-15.8]	4.25 [2.18-10.5]	2.17	↑	↑
	IDL ApoB-100	mg/dL	10.1 [8.14-12.4]	8.51 [8.14-12.4]	1.94 [1.07-5.22]	8.04	↑↑	↑↑
	LDL ApoB-100	mg/dL	60.5 [56.0-77.0]	57.5 [56.0-63.6]	61.1 [37.6-86.2]	113		
Lipoprotein composition	VLDL total lipids	mg/dL	117 [73.7-521]	106 [73.7-128]	72.0 [38.1-182]	29.5	↑	↑
	VLDL triglycerides	mg/dL	75.8 [45.7-394]	68.8 [45.7-82.8]	48.5 [25.2-123]	22.6		
	VLDL cholesterol	mg/dL	23.4 [16.2-53.3]	21.0 [16.2-25.7]	9.47 [4.33-27.9]	1.51	↑	↑
	VLDL free cholesterol	mg/dL	7.20 [3.53-28.6]	5.77 [3.53-8.62]	5.09 [2.72-12.8]	0		
	VLDL phospholipids	mg/dL	17.6 [11.8-73.6]	16.0 [11.8-19.2]	13.2 [6.29-30.9]	5.47		
	IDL total lipids	mg/dL	35.6 [24.0-127]	30.2 [24.0-40.9]	11.2 [6.82-41.9]	24.2	↑	↑
	IDL triglycerides	mg/dL	9.85 [4.35-69.4]	8.79 [4.35-10.9]	3.77 [0-19.4]	0	↑	↑
	IDL cholesterol	mg/dL	20.8 [14.1-30.4]	15.4 [14.1-26.2]	5.24 [0.57-13.6]	17.0	↑↑	↑↑
	IDL free cholesterol	mg/dL	5.83 [4.15-10.3]	4.38 [4.15-7.28]	1.44 [0.38-4.27]	4.69	↑	↑
	IDL phospholipids	mg/dL	5.54 [4.21-27.4]	5.17 [4.21-5.91]	3.23 [2.21-9.16]	7.24	↑	↑
	LDL total lipids	mg/dL	125 [119-142]	127 [119-142]	173 [99.5-235]	302	↓	↓
	LDL triglycerides	mg/dL	47.1 [44.1-54.0]	47.5 [46.7-54.0]	12.0 [8.32-20.0]	36.9	↑↑	↑↑
	LDL cholesterol	mg/dL	41.4 [35.9-52.3]	37.2 [35.9-52.3]	100 [55.9-138]	169	↓↓	↓↓
	LDL free cholesterol	mg/dL	21.3 [19.6-24.5]	20.2 [19.6-22.4]	28.8 [15.1-40.1]	48.5	↓	↓
	LDL phospholipids	mg/dL	35.8 [32.6-43.0]	35.9 [35.7-43.0]	56.9 [35.3-77.3]	95.7	↓	↓
	HDL total lipids	mg/dL	40.1 [34.1-46.9]	37.3 [34.1-43.0]	126 [106-161]	126	↓↓	↓↓
	HDL triglycerides	mg/dL	5.11 [2.06-13.6]	3.45 [2.06-6.77]	6.69 [5.13-11.1]	9.36		
	HDL cholesterol	mg/dL	12.3 [8.52-14.5]	12.9 [8.52-14.5]	53.7 [36.7-67.7]	45.5	↓↓	↓↓
	HDL free cholesterol	mg/dL	0 [0-0]	0 [0-0]	10.6 [4.99-14.7]	6.56	↓↓	↓↓
	HDL phospholipids	mg/dL	21.9 [18.8-25.1]	22.3 [18.8-25.1]	68.7 [58.4-88.3]	71.2	↓↓	↓↓

Full results can be found in Supplementary Table 2 and the supplemental materials. Values are given as median and range. Directions were graded as follows: ↑↑: all TD patients (ie, range) above the maximum of healthy controls; ↑: all TD patients higher than the median of healthy controls; ↓: all TD patients lower than the median of healthy controls; ↓↓: all TD patients below the minimum of healthy controls.

Abbreviations: ApoA-I, apolipoprotein A1; ApoB, apolipoprotein B; HDL, high-density lipoprotein; IDL, intermediate-density lipoprotein; LDL, low-density lipoprotein; NMR, nuclear magnetic resonance; TD, Tangier disease; VLDL, very low density lipoprotein.

Specifically, large and medium-sized VLDL (VLDL-2 and VLDL-3) carried higher concentrations of triglycerides, cholesterol, and phospholipids. Among LDL particles, all subtypes were rich in triglycerides while medium- and small-sized LDL (LDL-3-LDL-6) were depleted of cholesterol, free cholesterol, and phospholipids. LDL-4 was nearly undetectable in TD patients. HDL particles were depleted from cholesterol, free cholesterol, and phospholipids with markedly reduced particle numbers (ApoA-I). Full NMR results are available in the supplemental materials (17).

Interindividual differences among TD patients were most evident between patients #1 to #3 and patient #4, the latter having very high triglyceride levels (461 mg/dL), which also translated into altered composition of LDL subtypes [see supplemental materials for individual comparison and discussion section (17)]. Subgroup comparison of TD patients #1 to #3 is thus shown in Table 3.

**Figure 3.**
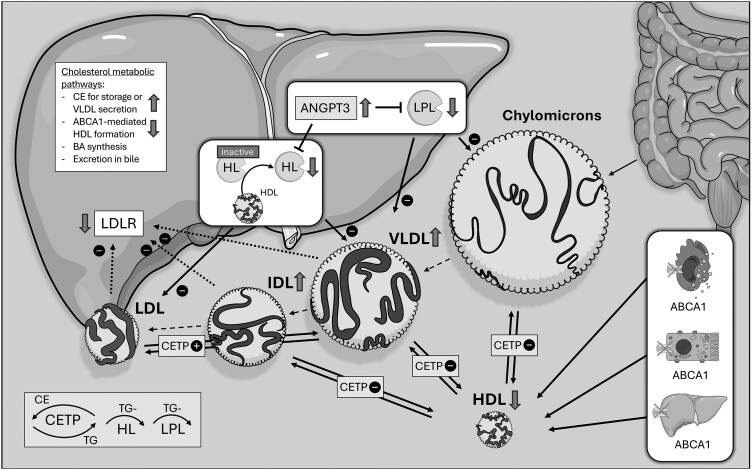
Summary of alterations in lipid metabolism in TD including hypotheses on potential underlying mechanisms. Grey arrows upwards indicate increased abundance or increased activity (enzymes) while arrows downwards indicate reduced abundance or decreased activity. Plus and minus indicate upregulated or downregulated pathways. In brief, ABCA-1 is expressed ubiquitously in the human body, with its activity in hepatocytes and enterocytes being mainly responsible for HDL production (right bottom of the picture) (4). Its expression on peripheral macrophages has been extensively investigated in the setting of reverse cholesterol transport and the associated cardiovascular benefits. Loss-of-function variants in ABCA1 lead to profoundly decreased levels of HDL and the impaired export of cholesterol from the above-mentioned cells, leading to the phenotype of severe hepatic steatosis and foam cell formation from macrophages that can infiltrate various organs including the intestinal tract (Fig. 1). The surplus of cholesterol/triglycerides in the liver increases VLDL secretion, especially as ABCA1-mediated HDL formation is impaired (19). VLDL from the liver and chylomicrons from the gut (ie, triglyceride-rich lipoproteins) are being lipolyzed (decrease in triglyceride content and increase in cholesteryl ester) through several mechanisms. Among them, cholesteryl ester transfer protein-mediated exchange of triglycerides for cholesteryl esters from LDL and HDL might be impaired in TD, as HDL is unavailable (20-22). Hepatic lipase is essentially involved in lipolysis of IDL and large-size LDL. Being stored inactively in hepatocytes (and to some extent bound to HDL), it is released from hepatocytes upon binding to HDL. As this mechanism is impaired in TD, HL activity could be lower and lipolysis of IDL and large-size LDL reduced (23, 24). ANGPTL3 is considered an inhibitor of LPL and HL and might be (independently) upregulated in TD, supporting the reduced activity of LPL and HL (25). Finally, hepatic steatosis might impair LDL-receptor-mediated endocytosis of LDL, IDL, and VLDL remnants (19). Abbreviations: ABCA1, ATP binding cassette transporter A1; ANGPTL3, angiopoietin-related protein 3; BA, bile acid; CE, cholesteryl ester; CETP, cholesteryl ester transfer protein; HDL, high-density lipoprotein; HL, hepatic lipase; IDL, intermediate-density lipoprotein; LDL, low-density lipoprotein; LDLR, low-density lipoprotein receptor; LPL, lipoprotein lipase; TD, Tangier disease; TG, triglycerides; TRL, triglyceride-rich lipoprotein; VLDL, very low density lipoprotein.

Hypotheses on the potential underlying pathophysiological mechanisms of the observed changes in lipid metabolism of TD patients are summarized in Fig. 3 and discussed later.

### Comparison With Second NMR Analysis

Another NMR spectroscopy analysis (method 2) including data from TD patients #1 and #3 is presented in Supplementary Tables 3 and 4 (17). In brief, the methods did not directly overlap with method 2, extending on particle size and chylomicrons while providing less granularity in terms of LDL subgroups. However, this analysis similarly indicated an increased abundance of chylomicrons, VLDL and IDL particles and a high triglyceride concentration in all lipoproteins including LDL. As blood samples from only 2 out of 4 TD patients were available and drawn at different timepoints, direct comparison was hampered. Correlations of overlapping parameters in healthy controls are shown in Supplementary Table 5 (17).

### Discrimination Between TD and Healthy Controls

**Figure 4.**
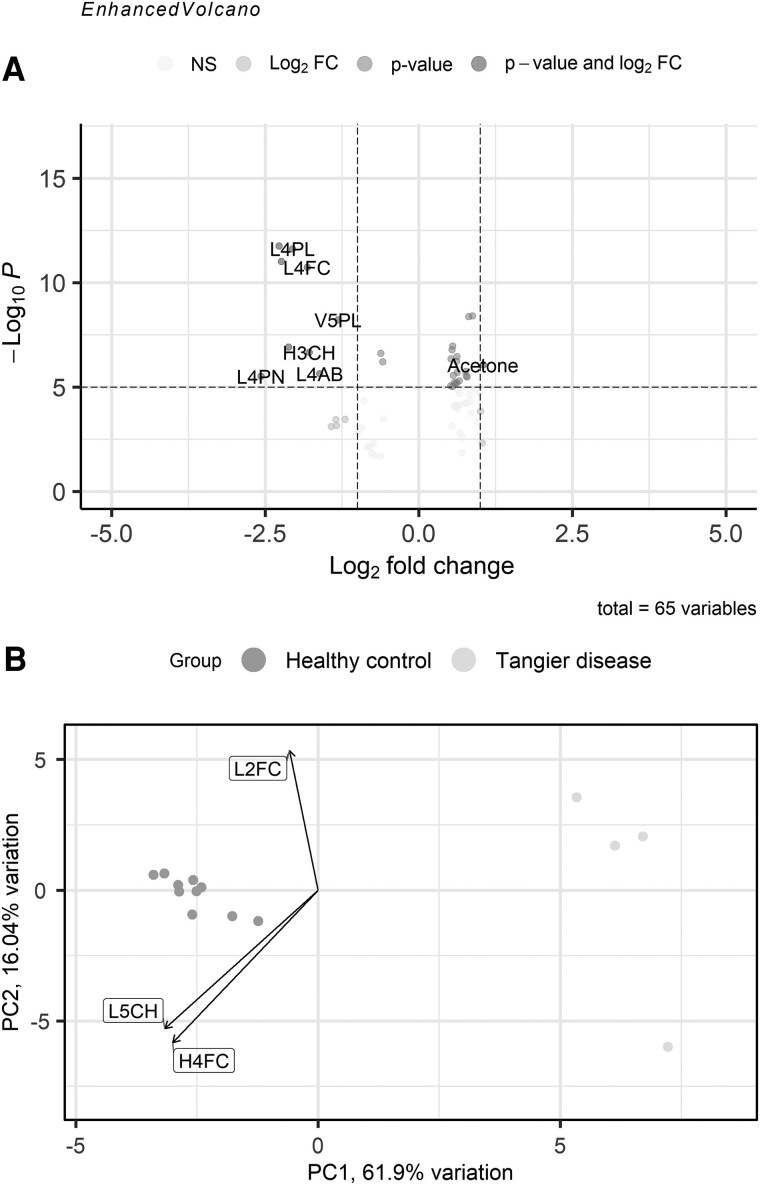
Volcano plot of metabolites concentrations in patients with TD (n = 4) and controls (n = 10) (A) and biplot following PCA (B). Detailed results of all differentially expressed metabolites (that could not be labeled) can be found in the supplemental materials (17). Abbreviations: logFC, log fold change; PCA, principal component analysis; TD, Tangier disease.

Finally, as an exploratory analysis, we analyzed the differential lipoprotein and metabolite abundance between TD and healthy controls using univariate statistics [Fig. 4A, Supplementary Fig. S5, Supplemental Materials (17)]. HDL and LDL-4 particle number and composition were among the most different from healthy controls. PCA confirmed the expected clear separation between TD and healthy controls with the differences in LDL-4 and HDL composition explaining most of the variance in principal component #1 (well separating TD vs healthy controls; Fig. 4B). Results excluding TD patient #4 were roughly similar [Supplementary Fig. S5 (17)]. Differential abundance and PCA from method 2 highlighted phospholipid concentrations in chylomicrons and large VLDL as decisive parameters [Supplementary Fig. S6 (17)].

## Discussion

In this case series, we describe the clinical presentation of 4 male patients with TD at different ages and with varying phenotypes including 2 novel pathogenic variants (c.2418G > A and c.5055.del), as discussed later. Also, we present comprehensive data on a profoundly altered lipid metabolism compared to healthy controls. Besides the pathognomonic depletion of HDL, our analysis describes several previously unknown consequences of *ABCA1* deficiency. While these data can only be hypothesis-generating, they potentially allow a better understanding of lipid metabolism through extensive characterization of *ABCA1* loss-of-function lipid phenotypes in humans.

First, VLDL particles were increased in number and larger in size (as specifically reported in method 2). This is usually considered to be associated with metabolic syndrome and insulin resistance, caused by both an exceeded adipose tissue storage capacity, adipose tissue dysfunction, and impaired peripheral triglyceride-rich lipoprotein (TRL) clearance but also an increased hepatic VLDL secretion (20, 26, 27). It is tempting to speculate that TRL clearance is impaired in TD, possibly due to reduced lipoprotein lipase (LPL) activity [previously reported in TD (28)], impaired storage capacity (eg, due to accumulation of foam cells in the adipose tissue), an increased secretion of VLDL by the liver as a compensatory mechanism in the presence of hepatic steatosis, or both. Previous reports that angiopoietin-related protein 3 (ANGPTL3), a potent inhibitor of LPL, is hypersecreted in TD models would support such a hypothesis (25). At the same time, an increased VLDL secretion from the liver has been suggested in a rodent model of TD and is a typical consequence of hepatic steatosis (29, 30).

The increased VLDL cholesterol concentration may be related to the overall higher number of VLDL particles or to the cholesteryl ester transfer protein (CETP)-mediated exchange of VLDL-triglycerides for LDL-cholesteryl esters (the latter being markedly decreased in our TD patients) during the longer circulation time in the absence of HDL particles (20, 31). This could also explain the marked enrichment of LDL particles with triglycerides evident in both methods.

Previously, inhibition of CETP by pharmacological therapies (primarily by blocking the exchange of HDL-cholesteryl ester and VLDL-triglycerides or LDL-triglycerides) resulted in a shift from small dense LDL to larger LDL particles, as LDL particles contain more triglycerides and less cholesterol (32, 33). Intriguingly, this situation might be similar to TD with respect to LDL lipid profiles, in which HDL is not available for a CETP-mediated exchange. This could, in turn, explain the abundance of larger LDL particles (LDL-1/LDL-2) and the depletion of small LDL particles but also the higher abundance of IDL particles (34).

In TD patients, the marked increase in IDL number and lipid content was surprising and might only partially be explained by the aforementioned reduced availability of CETP-mediated triglyceride exchange against HDL-cholesteryl ester (which would also explain the triglyceride enrichment of VLDL particles as seen in method 2). On the other hand, reduced hepatic lipase activity and/or overload could contribute to this phenomenon. While LPL acts on chylomicrons and VLDLs, IDLs are primarily lipolyzed by hepatic lipase (23). Furthermore, ANGPTL3, shown to be oversecreted in TD (25), has also been inversely associated with hepatic lipase activity in obese subjects (35). More importantly, hepatic lipase activity is essentially dependent on HDL [reviewed in (24)]. In brief, hepatic lipase is synthesized and stored predominantly by the liver in an inactive state but released by binding to HDL. This release appears to be controlled by HDL ApoA-II content (increased release) and apolipoprotein E content (inhibition), with the shift of the latter during fasting from HDL to TRL being responsible for mobilization of hepatic lipase (24, 36). Both HDL and ApoA-II are dramatically reduced in TD, explaining the reduced activity of hepatic lipase. In addition, a pool of hepatic lipase seems to be bound to HDL, which would also be impaired in TD (24). The observations of increased hepatic lipase in TD (28) upon heparin stimulation could in this regard represent an increased storage of inactive hepatic lipase (24).

Such a reduced hepatic lipase activity would also fit with the strikingly increased triglyceride content of LDL particles in both analyses [as previously shown in population-based studies and associated with cardiovascular risk (37)] and a reduced concentration of small dense LDL (LDL-3-LDL-6) in TD patients despite an increased prevalence of LDL-1/LDL-2 particles. Upon a CETP-mediated exchange of LDL-cholesteryl ester against VLDL-triglycerides (in triglyceride-rich/VLDL-rich environments), LDL particles would usually be expected to be lipolyzed by hepatic lipase, forming small dense LDL with their increased atherogenic potential [reviewed in (21)]. Reduced hepatic lipase activity could possibly explain this phenomenon in TD patients despite the presence of steatosis, which would rather be associated with increased small dense LDL (38). However, this could also be 1 explanation for the heterogeneous atherogenicity in TD that might be less pronounced than expected from lifelong lipid exposure (21, 39).

Finally, the LDL-receptor-mediated hepatic clearance of LDL may be impaired by overloaded cholesterol efflux through the VLDL pathway and disrupted efflux to HDL due to *ABCA1* loss-of-function (19). This might be in part mediated by a hepatic steatosis-associated reduced LDL uptake via the LDL receptor (40), potentially mediated by increased proprotein convertase 9 activity and subsequent LDL-receptor degradation (41-43).

In general, the NMR results from method 1 included data from patient #4 who had severely elevated triglyceride concentrations in the blood. We can exclude alcohol consumption as a potential reason, as patient #4 requires constant care by his wife, including complete support in terms of diet and eating. However, together with significantly limited physical activity, this could explain the altered lipid composition as compared to other TD patients, likely due to alimentary reasons. Especially at higher triglyceride levels, some evidence suggests a decreased TRL clearance together with CTEP-mediated triglyceride-transfer to LDL with subsequent lipolysis leading to the formation of small dense LDL, as evident in patient #4 (21, 39, 44). However, we have no evidence as to whether these lipid abnormalities are related to his TD manifestation or other causes.

Although homozygous *ABCA1* loss-of-function variants lead to no or an impaired plasma membrane localization of *ABCA1*, a reduced ApoA-I binding and lipid efflux to particles containing ApoA-I (ie, HDL subgroups), which in turn aggravate catabolism of ApoA-I (12 hours in TD vs > 60 hours in healthy controls) (45), plasma HDL levels are not necessarily zero but <10% of healthy controls (46). In line with this, we observed profoundly decreased but not unmeasurable HDL particles. While this might be due to methodological aspects, it seems that residual *ABCA1* activity is variable. This is in line with studies showing that lipid efflux (and corresponding HDL levels) as well as the functional consequences varied between different variants in *ABCA1* (46, 47). At the same time, it is not in line with a previous report stating that a TD patient exclusively had small pre-β_1_-HDL (48), likely explained by different genetic variants or methodologies.

Previous analyses of plasma lipoproteins reported unmeasurable total and large HDL levels as well as elevated LDL and small LDL concentrations in a patient with 3 variants in the *ABCA1* gene (49) and reduced numbers of VLDL (45% of controls) and LDL (69% of controls) at elevated IDL levels (136% of controls) in a compound heterozygote TD patient (50). While these studies only analyzed single TD patients, we observed median VLDL ApoB-100 corresponding to 312% of healthy controls (when calculated in a similar way), median IDL ApoB-100 of 521%, and LDL ApoB-100 of 99% (ie, comparable), especially IDL levels supporting our hypothesis of reduced hepatic lipase activity.

Although an accelerated atherogenicity might represent a serious complication in some patients with TD, evidenced by established cardiovascular diseases in approximately one-third of reported cases at the time of diagnosis (1), patient #3 had completely normal vasculature at the age of 47 including coronary arteries assessed by angiography, and patient #4 (age 77) was clinically asymptomatic. Importantly, the largest literature search on this topic in TD reported CAD in 24.8% and other vascular diseases in 21.8% (11).

Whereas the presence of chronic hyperferritinemia in these 2 TD patients with new variants cannot directly be attributed to TD, it likely indicates a low-grade inflammatory response to cholesterol deposition in macrophages, which has also been observed in other lipid-storage disorders such as Gaucher disease (51). As patient #3 had primarily an affection of the gastrointestinal tract and the heart, the first potentially considered a more immunogenic organ (as compared to vasculature alone), this might explain the pronounced chronic hyperferritinemia (>2000 ng/mL). Even though a primarily gastrointestinal manifestation of TD is not anticipated, it is in line with rare but emerging case reports on a predominant gastrointestinal manifestation of TD presenting with gastrointestinal bleeding (12) or chronic diarrhea (13, 52) or also asymptomatic clinical pictures (53). Interestingly, these reports only included male patients. While it was tempting to speculate on any sex differences in TD that potentially lead to different phenotypes and that have been described in the general population (27, 54), further studies are needed to understand the influence of sex on clinical phenotypes.

This study is limited by the sample size of TD being a rare disease. Thus, differences in lipid profiles must be seen as exploratory and must be validated in larger studies. Also, discussed mechanisms regulating lipid metabolisms must be regarded as hypotheses, as functional analyses were not available. Finally, a controversial discussion on the accuracy of NMR spectroscopy to assess specific lipid subclasses is ongoing (55, 56), but claims on its inaccuracy have not been substantiated despite medium-size LDL subfractions seem to be challenging (57). Nevertheless, NMR is a trusted and frequently applied methodology in lipid studies (58-60), showing good correlations with routine lipid measurements in multiple datasets (61, 62). As no reference method to describe lipoproteins in situ (ex vivo) exists so far, NMR spectroscopy probably best reflects in vivo conditions as minimal processing of serum samples is necessary before measurement. Importantly, while absolute numbers obtained from NMR spectroscopy should potentially be interpreted with more caution (56), we exclusively based our interpretations on relative concentrations compared to healthy controls.

In summary, the clinical presentation of TD might be heterogenous including gastrointestinal and neurological manifestations. Lipid metabolism in TD seems to be characterized by impaired LPL and especially hepatic lipase activity, potentially via increased ANGPTL3 secretion, and a CETP-mediated triglyceride flux toward IDL and predominantly large-sized LDL.

## Data Availability

Restrictions apply to the availability of some or all data generated or analyzed during this study to preserve patient confidentiality or because they were used under license. The corresponding author will on request detail the restrictions and any conditions under which access to some data may be provided. Preprocessed metabolomics data and scripts required to reproduce the figures are available via GitHub: https://github.com/viennaliverstudygroups/tangier-metabolomics.

## Abbreviations

ABCA1: ATP binding cassette transporter A1
ANGPTL3: angiopoietin-related protein 3
Apo-A: apolipoprotein-A
CAD: coronary artery disease
CETP: cholesteryl ester transfer protein
HDL: high-density lipoprotein
IDL: intermediate-density lipoprotein
LDL: low-density lipoprotein
LPL: lipoproteinlipase
NMR: nuclear magnetic resonance
PCA: principal component analysis
TD: Tangier disease
TRL: triglyceride-rich lipoprotein
VLDL: very-low-density lipoprotein
