# Correction to: Lipid Dysregulation in Tangier Disease: A Case Series and Metabolic Characterization

**DOI:** 10.1210/clinem/dgaf235

**Published:** 2025-04-18

This is a correction to: Georg Semmler, Clemens Baumgartner, Matthäus Metz (*et al*), Lipid Dysregulation in Tangier Disease: A Case Series and Metabolic Characterization, *The Journal of Clinical Endocrinology & Metabolism*, 2025; https://doi.org/10.1210/clinem/dgaf131

The following changes have been made to the originally published manuscript. Thomas Scherer has been marked as a co-corresponding author.

